# Connexin43 Mediated Delivery of ADAMTS5 Targeting siRNAs from Mesenchymal Stem Cells to Synovial Fibroblasts

**DOI:** 10.1371/journal.pone.0129999

**Published:** 2015-06-15

**Authors:** Shuo Liu, Corinne Niger, Eugene Y. Koh, Joseph P. Stains

**Affiliations:** Department of Orthopaedics, University of Maryland School of Medicine, Baltimore, Maryland, United States of America; Emory University School of Medicine, UNITED STATES

## Abstract

Osteoarthritis is a joint-destructive disease that has no effective cure. Human mesenchymal stem cells (hMSCs) could offer therapeutic benefit in the treatment of arthritic diseases by suppressing inflammation and permitting tissue regeneration, but first these cells must overcome the catabolic environment of the diseased joint. Likewise, gene therapy also offers therapeutic promise given its ability to directly modulate key catabolic factors that mediate joint deterioration, although it too has limitations. In the current study, we explore an approach that combines hMSCs and gene therapy. Specifically, we test the use of hMSC as a vehicle to deliver ADAMTS5 (an aggrecanase with a key role in osteoarthritis)-targeting siRNAs to SW982 synovial fibroblast-like cells via connexin43 containing gap junctions. Accordingly, we transduced hMSCs with ADAMTS5-targeting shRNA or non-targeted shRNA, and co-cultured them with synovial fibroblasts to allow delivery of siRNAs from hMSC to synovial fibroblasts. We found that co-culture of hMSCs-shRNA-ADAMTS5 and synovial fibroblasts reduced ADAMTS5 expression relative to co-culture of hMSCs-shRNA-control and synovial fibroblasts. Furthermore, ADAMTS5 was specifically reduced in the synovial fibroblasts populations as determined by fluorescence-activated cell sorting, suggesting transfer of the siRNA between cells. To test if Cx43-containing gap junctions are involved in the transfer of siRNA, we co-cultured hMSCs-shRNA-ADAMTS5 cells with synovial fibroblasts in which connexin43 was knocked down. Under these conditions, ADAMTS5 levels were not inhibited by co-culture, indicating that connexin43 mediates the delivery of siRNA from hMSCs to synovial fibroblasts. In total, our findings demonstrate that hMSCs can function as donor cells to host and deliver siRNAs to synovial fibroblasts via connexin43 gap junction in vitro. These data may have implications in the combination of hMSCs and gene therapy to treat diseases like osteoarthritis, in vivo.

## Introduction

Osteoarthritis (OA) is a degenerative joint disease that involves the interplay of numerous cell types, including articular chondrocytes and synovial fibroblasts, among others [1]. During OA, the production catabolic factors, such as matrix metalloproteinases (e.g., MMP-1, -3, -9 and -13) and aggrecanases (e.g., ADAMTS-4 and -5) by synovial fibroblasts and articular chondrocytes contribute to cartilage degradation [2–4]. The common end point for a patient suffering from OA is arthroplasty of the affected joint. While joint replacement technologies have advanced dramatically, there are still significant limitations to lifestyle with a reconstructed joint, as well as additional complication, including periprosthetic osteolysis, infection, and implant failure. Accordingly, there is a need to slow down the progression of joint destruction in patients with OA.

At least two promising therapies exist with the potential to alter the degenerative environment of the OA-joint, stem cell therapies with human mesenchymal stem cells (hMSCs) and targeted gene therapy. hMSCs are potent immunomodulators that can home to damaged tissue [5–7] and, thus, could offer therapeutic benefit in the treatment of arthritic diseases by suppressing inflammation and permitting tissue regeneration. However, these hMSCs must overcome the harsh catabolic environment of the OA joint. Likewise, gene therapy also offers therapeutic promise given its ability to directly modulate key catabolic factors that mediate joint deterioration. For example, genetic deletion of the aggrecanase ADAMTS5, which becomes elevated in the synovial fluid during OA, can prevent joint destruction in a murine model of surgically induced OA [8]. While a gene therapy based approach may be able to reduce the degenerative environment of the OA joint by suppressing catabolic genes, it has several limitations including difficulty maintaining the target gene in the joint compartment, difficulty maintaining sustained delivery and the inability to restore destroyed cartilage lesions. Similarly, the environment in the joint compartment during OA is such that a purely cellular approach (e.g., hMSC therapy) is likely to be influenced by and/or overwhelmed by the catabolic environment.

In the present study, we examine an approach that combines hMSCs and gene therapy to modulate gene expression in synovial fibroblasts-like cells. This approach is based on recent studies that have demonstrated that cells can communicate small RNAs (siRNAs, shRNAs or miRNAs) via gap junctions to adjacent cells, where they function to efficiently suppress gene expression with knockdown as high as 96% *in vitro* [9–14]. The data from these papers suggest that it is the processed, single stranded siRNAs, downstream of the DICER, that are being passed through gap junction channels. Indeed, up to 24-mers have been shown to pass through connexin43 (Cx43)-containing gap junctions [9]. Thus, gap junctions permit the exchange of siRNAs from a donor cell to a recipient cell and thus may represent a delivery vehicle for gene therapy.

Gap junctions are specialized communicative cell structures present in the plasma membrane of cells made up of connexin monomers that assemble on the plasma membrane of adjacent cells to create a transcellular channel. The resultant gap junction channel permits the direct exchange of small molecules among coupled cells, forming a functional syncytium for intercellular communication [15, 16]. Interestingly, Cx43 expression is increased in synovial fibroblasts and articular chondrocytes during OA [17–19]. Further, we and others have shown that inflammatory factors, which are present in the OA synovial fluid, increase Cx43 expression in both synovial fibroblasts and articular chondrocytes [20–24]. Accordingly, we wanted to examine if we could take advantage of this increase in Cx43 expression and intercellular communication in the OA joint to efficiently deliver siRNA-mediated gene therapy to cells of the joint, using hMSCs as a vehicle to both transport and deliver the siRNA as well as to serve an immunomodulatory role. Here, we test this concept using hMSC as a vehicle to deliver ADAMTS5-targeting siRNAs to SW982 synovial fibroblast-like cells via Cx43 containing gap junctions in vitro.

## Materials and Methods

### Ethics Statement

All animal procedures complied fully with the principles set forth in the "Guide for the Care and Use of Laboratory Animals" and were approved by the University of Maryland School of Medicine Office of Animal Welfare Assurances and Institutional Animal Care & Use Committee (Animal Welfare Assurance #A3200-01).

### Cell Culture

SW982 human synovial sarcoma cells (HTB-93, ATCC, Manassas, VA) were cultured in Leibovitz's L-15 medium (Cat. Number: 11415–064, Gibco; Grand Island, NY) with 10% fetal bovine serum (FBS, HyClone; Logan, UT) and 1% penicillin/streptomycin (Mediatech; Herndon, VA), and maintained in a 37°C incubator with atmospheric CO_2_ as described previously [25]. Human mesenchymal stem cells (hMSCs, PT-2501, Lonza, Allendale, NJ) were cultured in mesenchymal stem cell (MSC) growth medium (PT-3001, MSCBM, Lonza; Walksersville, MD) with manufacturer recommended growth supplement (PT-3001, BulletKit, Lonza), and maintained in a 37°C incubator with 5% CO_2_. Where indicated, cells were treated with 1ng/ml interleukin 1β (IL-1β, Calbiochem, La Jolla, CA) for 16 hours prior to harvest.

### Lentiviral transduction and siRNA transfection

shRNA lentiviral particles were purchased from Sigma-Aldrich (St Louis, MO), and siRNAs were purchased from Thermo Scientific (Waltham, MA). To verify the effectiveness of shRNA, SW982 cells were transduced with ADAMTS5-targeted shRNA lentiviral particles (SHCLNV, MISSION Lentiviral Transduction Particles, Sigma-Aldrich), negative control shRNA lentiviral particles (SHC002V, Mission Non-mammalian shRNA Control Transduction Particles, Sigma-Aldrich), or without viral particles, facilitated by ViraDuctin Lentivirus Transduction reagent (LTV-200, Cell Biolabs Inc., San Diego, CA) with manufacturer recommended protocol. Briefly, cells were seeded in 24-well plate with a density of 7.5 x 10^4^ cells/cm^2^. shRNA lentiviral particles were diluted with Leibovitz's L-15 medium, and then mixed with ViraDuctin transduction reagent to create a multiplicity of infection (MOI) of 20. The shRNA lentiviral mixture was incubated for 30 minutes at 37°C before adding into cells. Then, shRNA lentiviral mixture was added into SW982 cells, and incubated within Leibovitz's L-15 medium at 37°C with atmospheric CO_2_ overnight. shRNA-transduced cells were harvested to evaluate the expression of ADAMTS5 by western blot. SW982 cells were also transduced with RFP encoding lentiviral particles (SHC012, Mission pLKO.1-puro-CMV-TagRFP Positive Control Plasmid DNA, Sigma-Aldrich, St. Louis, MO) by the same protocol. RFP-transduced cells were used in the co-culture, followed by fluorescent activated cell sorting (FACS). hMSCs were transduced with ADAMTS5-targeted shRNA lentiviral particles (hMSCs-shRNA-ADAMTS5) or negative control shRNA lentiviral particles (hMSCs-shRNA-control) essentially as described above, except that shRNA lentivirus mixture was prepared with MSC growth medium. Transduction was performed in MSC growth medium with growth supplement at 37°C with 5% CO_2_. In addition, hMSCs were subjected to three rounds of sequential transduction with 24-hour interval to maximize the efficiency of transduction.

To knockdown Cx43, SW982 cells were transfected with human *GJA1*-targeted siRNA (ON-TARGETplus SMARTpool Human GJA1, Cat. Number: L-011042-00, Thermo Scientific Dharmacon, Waltham, MA). SW982 cells were seeded in 24-well plate with a density of 7.5 x 10^4^ cells/cm^2^. Then, SW982 cells were treated with human *GJA1*-targeted siRNA, facilitated with Lipofectamine 2000 (Invitrogen, Grand Island, NY) following modified manufacture protocol. Briefly, Lipofectamine 2000 reagent was diluted with Opti-MEM reduced serum medium (Gibco, Grand Island, NY). siRNA was also diluted with Opti-MEM and then mixed with diluted Lipofectamine 2000 reagent by 1:1 ratio in volume to create a final siRNA concentration of 0.25 pmol/μl. The mixture was incubated at room temperature for 5 minutes before adding to cells. Then, 200μl siRNA mixture was added into each well of cell culture plate, and incubated for 48 hours at 37°C with 5% CO_2._

### Immunohistochemistry

To determine the expression of Cx43 in vivo, immunohistochemistry was performed on paraffin sections of mice knee as we have described previously [26]. The intact knee joints from 8 week-old male C57BL/6 mice were harvested and fixed in 10% neutral buffered formalin, followed by decalcification in 14% EDTA. The decalcified specimens were embedded in paraffin, sectioned coronally using a microtome, and mounted on microscope slides. Immunohistochemistry was performed on paraffin embedded sections using the Vectastain Elite ABC kit and DAB Peroxidase substrate kit (Vector Labs; Burlingame, CA). Rabbit anti-Cx43 antibodies (1:1000) were purchased from Sigma (Cat. Number C6219, St Louis, MO,). A non-immune rabbit IgG (Cat. Number 31235, Thermo Scientific, Waltham, MA) was used as a negative control.

### RNA isolation and Quantitative RT-PCR

Cells were harvested for RNA extraction using Directzol RNA miniprep (Zymo Research, Irvine, CA). RNA (1μg) was reverse transcribed with either iScript (Bio-Rad, Hercules, CA) or RevertAid (Fermentas, Thermo Scientific, Waltham, MA) reverse transcription master mix, according to the manufacturers instructions. Quantitative real time PCR was carried out using the SYBR green method, as described previously [1]. The relative gene expression was simultaneously normalized to the expression of three house keeping genes, *RPL13*, *HPRT* and *GAPDH* using the GeNorm v3.5 Software (Ghent University Hospital Ghent, Belgium), as described previously [27]. The primer sets used for PCR are shown in Table 1.

**Table 1:** PCR Primer pairs for quantitative real time RT-PCR.

Target	Primer 1	Primer 2
*GJA4/*Cx37	ATT AAG GAG CAT GGC GAC TTC T	CCC AGG AGG AAA AGC ATG AG
*GJA5*/Cx40	ACA CCA CCC CCC GAC TTT A	TTA TTG CTG AAG GGA TTG AAG AAT T
*GJA1*/Cx43	TTT GAT GGA CCT GGA GGA AAT C	TGA GCA TCC CCT CCA ATA CC
*GJC1*/Cx45	AAT GCT AAG ATC GCC TAC AAG CA	CTC CTC ATG GCT GCC ATA CTG
*GJA3*/Cx46	CAA CAC GGT GGA CTG CTT CA	AGG CCA CCG CCA GCA T
*ADAMTS5*	AAT AAC CCT GCT CCC AGA AAC A	GCG GTA GAT GGC CCT CTT C
*HPRT*	TGA CAC TGG CAA AAC AAT GCA	GCT TGC GAC CTT GAC CAT CT
*GAPDH*	CCC ACT CCT CCA CCT TTG AC	CAT ACC AGG AAA TGA GCT TGA CAA
*RPL13*	AGC CTT CGC TAG TCT CCG TAT G	TGG CTC TTT TTG CCC GTA TG

### Western Blotting

Whole cell extracts were harvested from adherent cells. Proteins were solubilized in 300 μl/well modified RIPA buffer (50 mM Tris pH 8.0, 150 mM NaCl, 1.0% NP-40, 0.5% sodium deoxycholate, 0.1% SDS; completed with 10 mM sodium pyrophosphate, 10 mM β-glycerophosphate, 10 mM sodium fluoride, 1 mM EDTA, 1 mM EGTA, 1 mM sodium orthovanadate and 1X protease and phosphatase inhibitor cocktail). The samples were sonicated. Insoluble material ware removed by centrifugation. The supernatants were stored at −20°C until use. Sample were subjected to separation on 10% SDS-PAGE gels and then transferred to polyvinylidene difluoride membranes (Millipore; Bedford, MA). Subsequently, membranes were blocked in 5% non-fat dry milk, and were incubated with primary antibodies overnight, with the horseradish peroxidase-conjugated secondary antibodies (GE Healthcare Bio-Sciences; Picataway, NJ). Membranes were visualized using SuperSignal West Dura Extended Duration Substrate (Thermo Scientific), and imaged with ChemiDoc MP system (Bio-Rad). The signal was quantified by Image Lab software (Bio-Rad).The rabbit anti-Cx43 antibody was purchased from Sigma (1: 1000, Cat. Number C6219). The mouse anti-GAPDH antibody was purchased from Millipore (1:2000, Cat. Number MAB374). The rabbit anti-ADAMTS5 (1:1000) antibody was purchased from Genetex (Cat. Number GTX100332, Irvine, CA,). Gels were quantified by densitometry using ImageJ. Data are shown relative to GAPDH.

### Serotonin Gap Junctional Coupling Assay

To demonstrate intercellular communication between SW982 cells and hMSCs, we utilized a previously reported gap junctional communication assay [28]. Briefly, hSERT-transfected SW982 cells (donor cells) and hMSCs (recipient cells) were co-cultured on a 10 mm coverslips in a 24-well plate at a 1: 4 ratio. The cells were maintained in MSC growth medium (PT-3001, MSCBM, Lonza; Walksersville, MD) with manufacturer recommended growth supplement (PT-3001, BulletKit, Lonza) in a 37^°^C incubator with 5% CO_2_. 24 hours after co-culture, cells were incubated in serum free α-MEM medium containing 400 nM serotonin for 20 minutes under 37°C. After rinsing with 1X HBSS for 3 times, the co-cultured cells were fixed in 4% paraformaldehyde for 15 minutes at room temperature, followed by permeabilization with 0.2% Trition-x-100 for 2 minutes. Then, co-cultured cells were incubated with blocking buffer (10% goat serum and 0.2% Tween 20 in 1X HBSS) for 1 hour at room temperature. Immunohistochemistry staining was performed on coverslips. Mouse anti-SERT antibody (1:1000, MAB5618, Millipore, Billera, MA) and rabbit anti-serotonin antibody (1:1000, S5545, Sigma-Aldrich, St Louis, MO) in blocking buffer were incubated with coverslips for 1 hour at room temperature, followed by incubation with anti-mouse Cy3 (1:500) and anti-rabbit Alex 488 (1: 500) antibodies in blocking buffer for 1 hour. Stained coverslips were mounted onto slide with Fluoromount aqueous mounting medium (F4680, Sigma-Aldrich, St Louis, MO) and imaged with fluorescent microscope.

### hMSC and SW982 Co-culture experiments

To setup co-culture, shRNA-transduced hMSCs and SW982 cells were mixed at 2 to 1 ratio with a total seeding density of 5 x 10^4^/cm^2^ in 24-well plate, based on previous reports [9, 29]. Briefly, transduced or transfected cells were washed in Hank’s balanced saline solution, trypsinized, pelleted by centrifugation and then re-suspended in corresponding growth media. Cell counts were performed and the cells were mixed as indicated. The mixed cell populations were cultured in MSC growth medium (PT-3001, MSCBM, Lonza) with manufacturer recommended growth supplement (PT-3001, BulletKit, Lonza) in a 37^°^C incubator with 5% CO_2_ until the cells were harvested 48 hours later. Three sets of co-culture were tested: 1) Co-culture of hMSCs-shRNA-ADAMTS5 and SW982 cells was setup to test whether hMSCs can deliver shRNA to recipient synovial fibroblasts to knock down ADAMTS5 expression. Co-culture of hMSCs-shRNA-control and SW982 cells was setup as control. 2) Co-culture of hMSCs-shRNA-ADAMTS5 and SW982-siRNA-Cx43 cells was setup to test whether delivery of shRNA is mediated by gap junction Cx43. Co-culture of hMSCs-shRNA-ADAMTS5 and SW982-siRNA-control cells was setup as control. 3) Co-culture of hMSCs-shRNA-ADAMTS5 and SW982-RFP cells was setup to test whether the delivered shRNA knocks down the expression of ADAMTS5 in recipient synovial fibroblasts. Co-culture of hMSCs-shRNA-control and SW982-RFP cells was setup as control. All the co-cultures were incubated with 1ng/ml IL-1β for 16 hours before ending the co-culture at 48 hours. This was done to maximize the expression of ADAMTS5 an the synovial cells. Cells were either directly lysed to prepare for western blot, or RFP-positive SW982 cells were isolated by FACS, and then were prepared for quantitative real-time RT-PCR.

### Fluorescent Activated Cell Sorting

Cells were sorted for RFP with a two-laser FACSAria (BD Biosciences) cell sorter. RFP-positive events were determined based on RFP-negative control cells. RFP-positive cells were sorted directly into TriPure RNA isolation reagent (Roche, Indianapolis, IN) for subsequent RNA isolation, reverse transcription and real time PCR.

### Statistics

All statistical analyses were conducted using Prism 6 software (GraphPad, LaJolla, CA). Two-tailed unpaired Student's t-test were performed, as appropriate. A p value ≤ 0.05 was considered significant.

## Results

### Cx43 is abundantly expressed in joint and in hMSCs and synovial fibroblasts in culture

**Fig 1:**
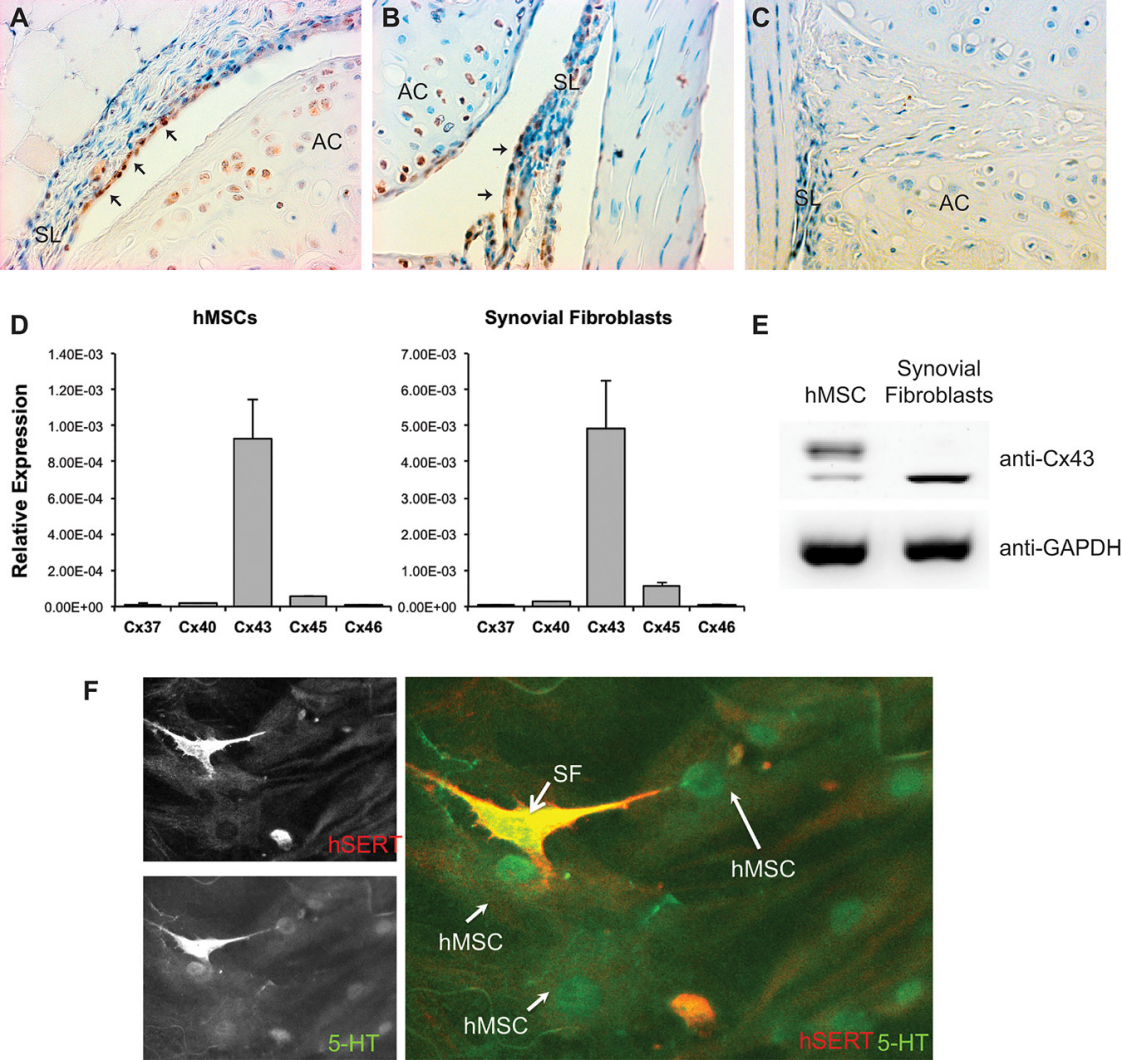
Cx43 is robustly expressed in synovial fibroblasts and hMSCs. (A-B), Sections of murine knee joint were labeled with anti-Cx43 antibodies (brown). Cx43 is detected in the synovial fibroblasts (arrows) in the synovial lining (SL), as well as in the articular chondrocytes in the articular cartilage (AC). (C), A sections of murine knee joint were labeled with non-immune IgG as a negative control. (D), Quantitative real time RT-PCR was performed on RNA isolated from hMSCs and synovial fibroblasts in culture using primer pairs for the genes encoding Cx37, Cx40, Cx43, Cx45 and Cx46. Histograms indicate means ± standard deviation. (E), A western blot of whole cell extracts from hMSCs or synovial fibroblasts probed with anti-Cx43 antibodies reveals expression of Cx43 in both cell types. The expression of Cx43 in hMSCs is composed of a larger percentage of the higher molecular weight phosphorylated Cx43 than in SW982 cells, which express mostly the non-phosphorylated form. The images are from non-contiguous lanes of the same blot and exposure. GAPDH abundance was used as a loading control reference. (F) Immunofluorescnece microscopy reveals that hSERT+ SW982 cells (red) can take up serotonin/5-HT (green) and communicate it with hMSCs, indicating functional gap junctional communication between the two cell types. A representative image is shown.

To determine the expression of Cx43 in the knee joint, immunohistochemistry was performed on paraffin sections of healthy mouse knees. Our immunohistochemistry staining demonstrated that Cx43 is abundantly expressed by synovial fibroblasts and articular chondrocytes (Fig 1A–1C). To verify that Cx43 is the predominant connexin expressed by hMSCs and synovial fibroblasts (SW982) in vitro, we measured the expression of Cx37, Cx40, Cx43, Cx45, and Cx46 in both cell lines by quantitative real-time RT-PCR. The result demonstrated that in both hMSCs and synovial fibroblasts (SW982) the level of *GJA1*/Cx43 is much higher than the other connexins gene typically associated with cells of the mesenchymal origins (Fig 1D). This finding was confirmed by our western blot data, which demonstrated that Cx43 is expressed in both hMSCs and SW982 cells (Fig 1E).

Using a recently developed cell-to-cell communication assay [28], we showed that hSERT+-SW982 (red) cells form gap junctions capable of communicating serotonin (5-HT, green) to hMSCs in co-culture (Fig 1F), indicating functional gap junction channels forming between the two cell types. 5-HT was not detected in either cell type in the absence of transfection of the SW982 cells with hSERT expression vector (data not shown).

### ADAMTS5 shRNA down regulated target gene expression in synovial fibroblasts

**Fig 2:**
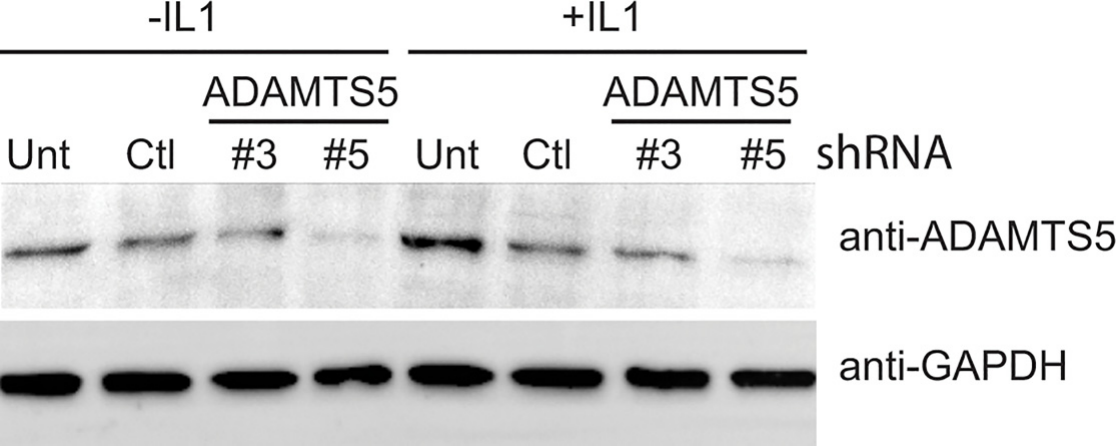
Effective knockdown of ADAMTS5 in SW982 synovial fibroblasts cells transduced with ADAMTS5 targeting shRNA lentiviral particles. SW982 cells were untransduced (unt), or transduced with non-targeting shRNA (Ctl) or with two distinct ADAMTS5-targeting shRNA (denoted #3 or #5). Post-transduction the cells were treated in the presence or absence of IL-1β (1ng/ml, 16h) to induce ADAMTS5 expression. Subsequently, a western blot of whole cell extracts from these cells were run and probed with anti-ADAMTS5 or anti-GAPDH antibodies as indicated. GAPDH abundance was used as a loading control reference. A representative blot is shown.

To determine the effectiveness of our shRNA, ADAMTS5-targeted shRNA lentiviral particles were transduced into SW982 cells with or without stimulation of IL-1β. Two different ADAMTS5 shRNA encoding lentiviral constructs were used to transduce the cells (denoted #3 and #5, respectively). Fourty-eight hours post-transduction, SW982 cells were then prepared for western blot analysis. ADAMTS5 shRNA construct #5 was substantially more effective at reducing both the basal and IL-1β stimulated levels of ADAMTS5 protein relative to the scrambled control construct (Fig 2). Accordingly, construct #5 was used for the remainder of the study.

### Co-culture hMSCs-shRNA-ADAMTS5 with synovial fibroblasts resulted in effective ADAMTS5 knockdown

**Fig 3:**
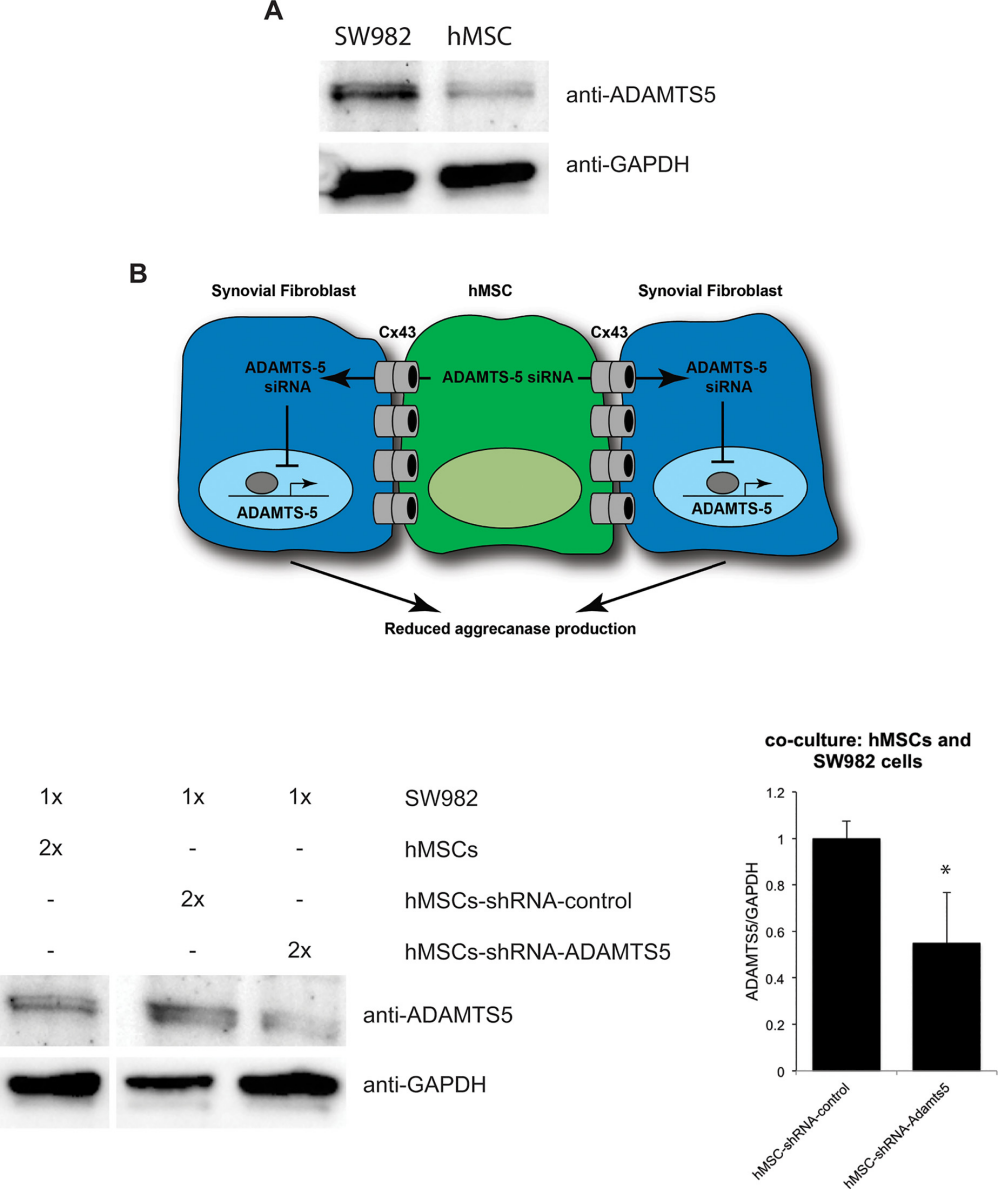
Co-culture of hMSCs transduced with ADAMTS5-targeting shRNA can effectively knockdown the expression of ADAMTS5 in SW982 cells. (A), A western blot of whole cell extracts from SW982 cells or hMSCs in mono-culture probed with anti-ADAMTS5 or anti-GAPDH antibodies, as indicated, shows that ADAMTS5 is most abundantly expressed in SW982 cells, although a modest amount is also observed in hMSCs. A representative blot is shown. (B), A cartoon depiction of the proposed model in which hMSCs, which have been “pre-loaded” with ADAMTS5-targeting shRNA, are co-cultured with synovial cells. These cells are capable of forming Cx43- containing gap junctions. These gap junction channels permit the communication of the siRNA from the hMSCs to the synovial cells, resulting in an attenuation of the production of ADAMTS5 in the recipient synovial cell. (C) A representative western blot is shown for co-cultured hMSCs and SW982 synovial fibroblasts. The hMSCs have been previously transduced with non-targerting control shRNA (hMSCs-shRNA-control), ADAMTS5-targeting shRNA (hMSCs-shRNA-ADAMT5), or left untransduced (hMSCs). The cells are co-cultured at the indicated ratio (2x hMSCs to 1x SW982 cells). The blot was probed with anti-ADAMTS5 or anti-GAPDH antibodies, as indicated. The images are from non-contiguous lanes of the same blot and exposure. The graph to the right reveals the combined data from quantitation of band intensity from 4 independent replicates of the experiment. Histograms indicate means ± standard deviation. *, indicates a p-value <0.05 relative to control.

In order to test our strategy that ADAMTS5-targeting shRNA can be delivered from donor hMSCs to recipient synovial fibroblasts, we transduced hMSCs with shRNA, and then co-cultured them with synovial fibroblasts, followed by western blot analysis. In addition, we also measured the expression of Cx43 in both hMSCs and synovial fibroblasts individually by western blots (Fig 3A). In monoculture, we observed that SW982 synovial fibroblasts expressed significantly more ADAMTS5 than hMSCs. Co-cultures of hMSCs-shRNA-ADAMTS5 and SW982 (n = 4) expressed less ADAMTS5 compared to co-culture of hMSCs-shRNA-control and SW982 (n = 4, p<0.05, Fig 3B), suggesting that perhaps ADATMTS5 shRNA were transferred from hMSCs to the target SW982 cells. However, since hMSCs express low levels of ADAMTS5 we could not fully rule out that this reduction in gene expression was caused by knockdown of the endogenous ADAMTS5 in the hMSCs.

**Fig 4:**
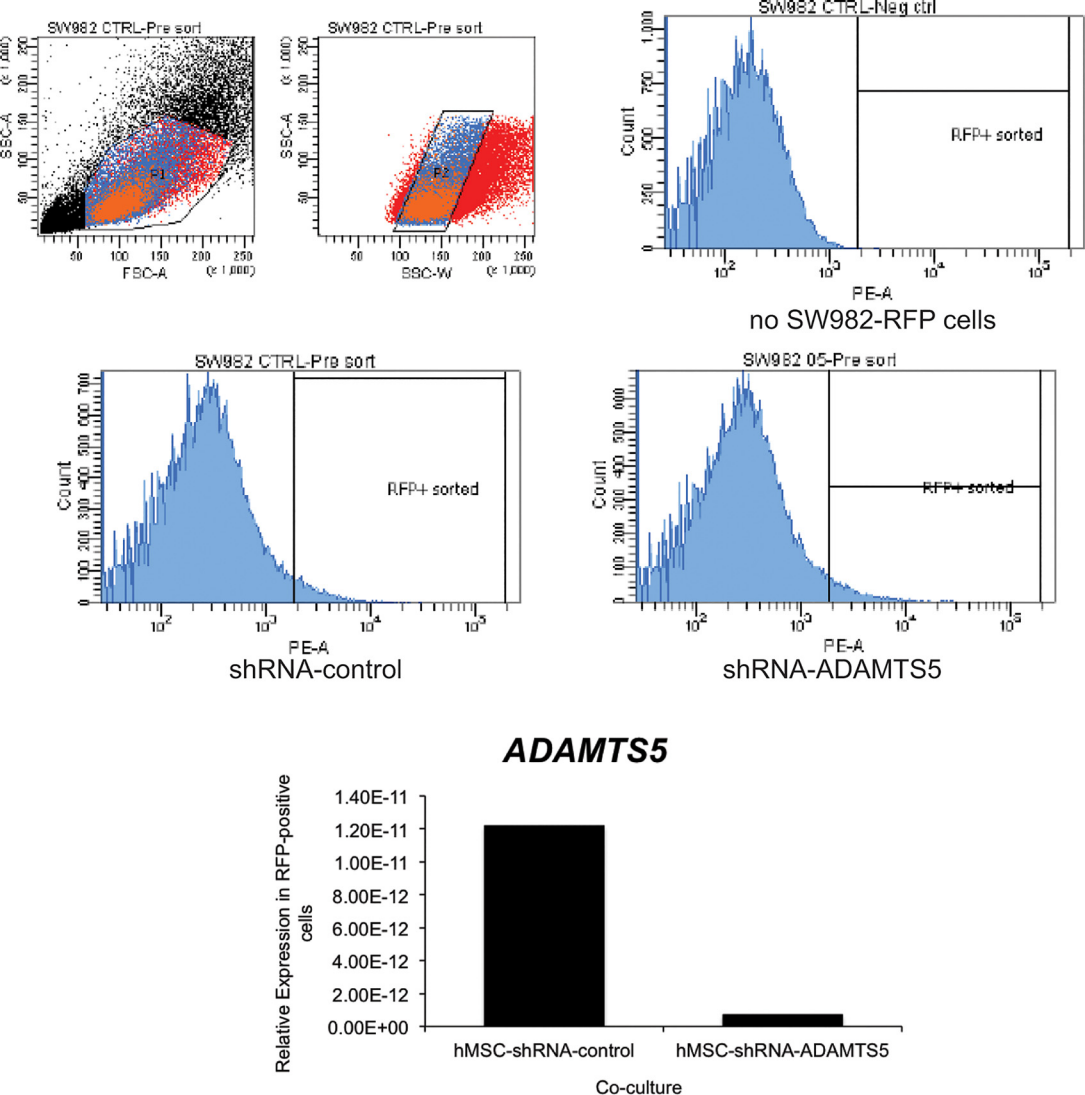
Co-culture of hMSCs transduced with ADAMTS5-targeting shRNA effectively reduces ADAMTS5 expression in flow cytometry sorted RFP-labeled SW982. (A) Following co-culture of non-targeting control shRNA or ADAMTS5-targeting shRNA transduced hMSCs with RFP labeled SW982 cells, the cells were sorted by flow cytometry. The gating threshold (RFP+ sorted) was set based on the analysis of co-cultured hMSCs with unlabeled SW982 cells (no SW982-RFP cells). RFP-positive SW982 cells that were co-cultured with hMSCs previously transduced with non-targeting control-shRNA or ADAMTS5-targeting shRNA were sorted directly into TriPure reagent for RNA isolation. (B) The histogram shows the relative gene expression for ADAMTS5 in the RFP-positive cell population isolated after co-culture with hMSC-shRNA-control or hMSC-shRNA-ADAMTS5 cells as determined by quantitative real time RT-PCR. The result is from a representative experiment.

In order to clarify this point, we performed the co-culture experiments with RFP-labeled SW982 cells, and then isolated the RFP-positive SW982 cells from the hMSCS by FACS. Then, we determined the level of ADAMTS5 in these purified SW982 cells by quantitative real-time RT-PCR. As was observed in the previous co-culture experiments, RFP-positive SW982 cells that were co-cultured with hMSCs-shRNA-ADAMTS5 express much less ADAMTS5 mRNA than RFP-positive SW982 cells that were co-cultured with hMSCs-shRNA-control (Fig 4). Because SW982 cells did not contain any shRNA before co-culture, decreased ADAMTS5 in SW982 is most likely due to shRNA delivered from donor hMSCs. Therefore, this finding indicates that hMSCs can act as a vehicle to host and deliver shRNA to surrounding recipient cells and to effectively knock down the expression in the recipient cells.

### Cx43 mediated the delivery of shRNA from hMSC to synovial fibroblasts

**Fig 5:**
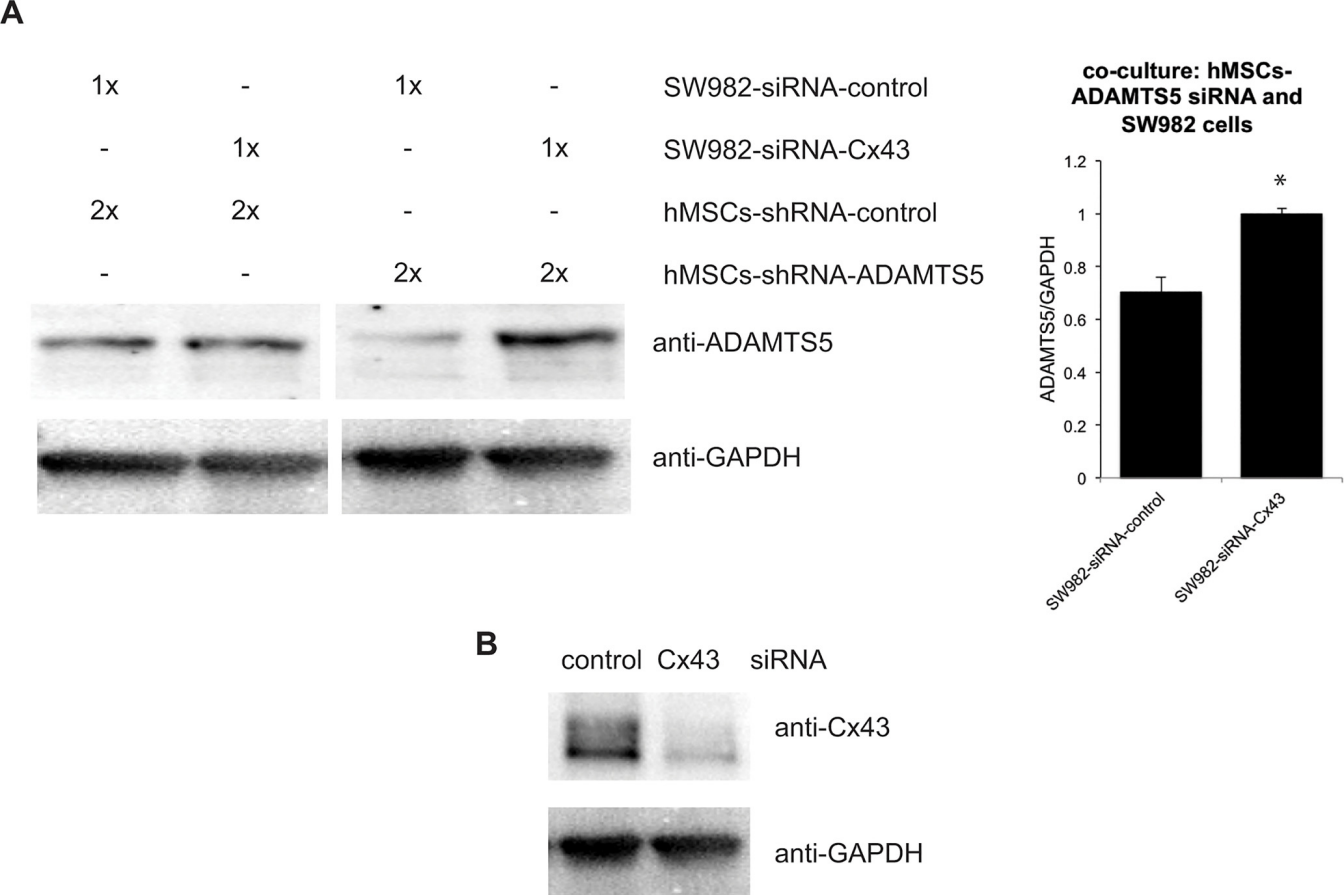
Cx43 mediated the delivery of shRNA from hMSC to synovial fibroblasts. (A), Western blot from co-culture of SW982 cells transduced with Cx43-targeting siRNA (SW982 with less Cx43) or non-targeting control siRNA and hMSCs transduced with ADAMTS5-targeting shRNA or non-targeting control shRNA. Knockdown Cx43 on SW982 cells preserved the expression of ADAMTS5 in the co-culture of SW982 cells and ADAMTS5-targeting shRNA transduced hMSCs. The graph to the right reveals the combined data from quantitation of band intensity from 3–4 independent replicates of the experiment. The histogram indicates mean ± standard deviation. *, indicates a p-value<0.05 relative to control. (B), Western blot of Cx43 in SW982 cells transduced with Cx43-targeting siRNA or non-targeting control siRNA: The expression of Cx43 in SW982 cells decreased by transduction of Cx43-targeting siRNA.

Previous studies have implicated Cx43 containing gap junctions in the transfer of siRNA between cells [9, 10]. Thus, to examine that Cx43 participates in the delivery of siRNA from hMSC to synovial fibroblast, we repeated the previous co-culture by using synovial fibroblasts with deficient Cx43. First, we transfected SW982 cells with Cx43-targeted siRNA to knock down the expression of Cx43 (SW982-siRNA-Cx43) or with a non-targeting siRNA control (SW982-siRNA-Control). Then, we co-cultured the SW982-siRNA-Cx43 (deficient Cx43) with hMSCs-shRNA-ADAMTS5 (n = 3), and compared it to co-culture of SW982-siRNA-control (intact Cx43) and hMSCs-shRNA-ADAMTS5 (n = 4). Western blots showed that in the absence of ADAMTS5 shRNA, knockdown of Cx43 in the SW982 cell population had no effect on ADAMTS5 expression (p<0.01, Fig 5A). Conversely, when ADAMTS5-shRNA expressing hMSCs were co-cultured with SW982 that had been transduced with non-targeting siRNA (SW982-siRNA-Control), ADAMTS5 expression was markedly reduced. This effect was completely abrogated in SW982-siRNA-Cx43 cells. This result indicates that loss of Cx43 expression by synovial fibroblasts blunts the ability of hMSCs expressing ADAMTS5 targeting shRNA from down regulating ADAMTS5 in the synovial cell population, suggesting a significant role for Cx43 in the delivery of siRNA from hMSCs to synovial fibroblasts.

## Discussion

In the current study, we tested a novel siRNA delivery strategy, which uses hMSCs as donor cells to host and deliver siRNA to recipient synovial fibroblasts via Cx43-conatining gap junctions in order to knock down ADAMTS5, a gene associated with OA pathogenesis, in recipient synovial fibroblasts. Our findings demonstrate that siRNA could be delivered from hMSCs to the interior of targeted synovial fibroblasts through an intercellular gap junction channel composed of connexin43. In addition, the delivered shRNA successfully down regulates the targeted gene (ADAMTS5) in recipient synovial fibroblasts. While a small number of papers have shown that Cx43 can mediate the transfer of siRNA or other small RNA species [9–14], this is the first demonstration of the effectiveness of siRNA transfer between hMSCs to synovial cells.

The implications of the use of cell-based delivery systems for gene therapy have been documented [30]. For example, pre-loading the hMSCs with siRNA will enhance the retention of the siRNA in the host tissue and will decrease the concentration of siRNA needed to effect host cell function, as direct injection of siRNA would be subjected to a dilution effect from host tissue and would more readily diffuse out of the joint compartment. Further, the use of autologous hMSCs to deliver the siRNA avoids complications from direct administration of nucleic acids or viral particles into the body fluid and the subsequent impact of the immune system.

For OA, the use of this technology is potentially quite appealing for several reasons. As we mentioned previously, Cx43 expression in synovial fibroblasts and articular chondrocytes is markedly increased during OA [17, 18]. As the transfer of siRNA between the hMSC and the host tissue is, at least in part, Cx43-dependent, the upregulation of Cx43 in the joint tissue during OA may lend itself very well to the delivery of siRNA. Indeed, our results demonstrate strikingly efficient knockdown of ADAMTS5 from hMSCs to synovial cells. Another advantage of this system is that the degenerating joint is likely to also attract and retain hMSCs, which preferentially home to damaged tissue [31, 32]. Once in the joint, hMSCs are likely to improve cartilage repair and ameliorate inflammation. In vitro, hMSCs have been shown to enhance the function of osteoarthritic articular chondrocytes in micromass culture, increasing proteoglycan and extracellular matrix production and suppressing *MMP13* expression [33]. In addition, hMSCs have chondrogenic potential in vivo and in the correct context may be able to contribute directly to tissue repair [34–36].

For the hMSCs to be effective at modulating the joint for tissue regeneration/repair, it is imperative that the cells be able to attenuate the catabolic environment of the joint. In a murine model of surgically induced OA, ADAMTS5 is absolutely required for disease progression and joint degeneration [8]. Thus by suppressing ADAMTS5 via siRNA-mediated gene knockdown, we postulate that this could buy time for tissue repair or at least slow down tissue catabolism and joint degeneration. The present study sets the stage to test the feasibility of such a therapeutic approach in vivo.

Additionally, once the siRNA are delivered from the hMSCs to the host tissue, it is possible that the host tissue itself could propagate the siRNA among themselves via gap junctions, leading to a change in tissue function as a result of a subset of cells. Indeed, not only are synovial cells coupled by Cx43 containing gap junctions, as we have shown here, but articular chondrocytes may also be coupled by extensive gap junction networks [37]. If this gap junction network functions in articular cartilage, then this could potentially facilitate penetration of the siRNA throughout the articular cartilage, as the cell-to-cell communication among articular chondrocytes could circumvents the steric hindrance of dense tissues like cartilage. Future studies will examine these possibilities in articular chondrocyte cells and using *in vivo* and *ex vivo* approaches.

A limitation of the study is that the contribution of synovial derived ADAMTS5 to articular cartilage destruction in OA is not clear. While synovial produce ADAMTS5 and ADAMTS5 levels in the synovial fluid are high in OA, several cell types in the joint can express ADAMTS5 [38–40]. However, synovial but not articular chondrocyte expression of ADAMTS5 has been demonstrated following inflammatory cytokine treatment in a murine knee joint explant model [40]. It is becoming increasingly clear that OA is a disease of the total joint and a more holistic approach of targeting numerous cell types may be essential to mitigating joint degeneration. The broad distribution of Cx43 expression among the various cells of the joint make this approach intriguing if the delivery is generalizable to other cells in addition to synovial fibroblasts.

While the present study demonstrates the feasibility of using MSCs to target synovial gene expression in a Cx43-dependent manner, effective use of this technology for disease management context will likely require the delivery of a cocktail of siRNAs, as regulation of a single gene will likely be ineffective at attenuating disease progression. Future studies will explore this possibility in OA models.

In summary, our findings demonstrate that siRNA could be delivered from hMSCs to synovial fibroblasts in a connexin43-dependent manner. In addition, the delivered siRNA successfully down regulates the targeted gene (ADAMTS5) in recipient synovial fibroblasts. Since ADAMTS5 has been proved to be a key catabolic factor in OA, findings in current study suggest that hMSCs carrying ADAMTS5-targeted shRNA could have great potential in eliminating the catabolic effect of osteoarthritic cells in addition to its regeneration of articular chondrocyte. Furthermore, since Cx43 is widely expressed in different types of cells, this siRNA delivery strategy could be extended to any hMSCs-related therapy to down regulate various pathological genes in different recipient cells.
